# Effects of biosurfactants on the viability and proliferation of human breast cancer cells

**DOI:** 10.1186/s13568-014-0040-0

**Published:** 2014-04-15

**Authors:** Cristina Duarte, Eduardo J Gudiña, Cristovao F Lima, Ligia R Rodrigues

**Affiliations:** 1 CEB - Centre of Biological Engineering, University of Minho, Braga 4710-057, Portugal; 2 Department of Biology, CITAB - Centre for the Research and Technology of Agro-Environmental and Biological Sciences, University of Minho, Braga 4710-057, Portugal

**Keywords:** Surfactin lipopeptide, Lactobacillus paracasei glycoprotein, Cell viability, Cell cycle, Breast cancer

## Abstract

Biosurfactants are molecules with surface activity produced by microorganisms that can be used in many biomedical applications. The anti-tumour potential of these molecules is being studied, although results are still scarce and few data are available regarding the mechanisms underlying such activity. In this work, the anti-tumour activity of a surfactin produced by *Bacillus subtilis* 573 and a glycoprotein (BioEG) produced by *Lactobacillus paracasei* subsp. *paracasei* A20 was evaluated. Both biosurfactants were tested against two breast cancer cell lines, T47D and MDA-MB-231, and a non-tumour fibroblast cell line (MC-3 T3-E1), specifically regarding cell viability and proliferation. Surfactin was found to decrease viability of both breast cancer cell lines studied. A 24 h exposure to 0.05 g l^-1^ surfactin led to inhibition of cell proliferation as shown by cell cycle arrest at G1 phase. Similarly, exposure of cells to 0.15 g l^-1^ BioEG for 48 h decreased cancer cells’ viability, without affecting normal fibroblasts. Moreover, BioEG induced the cell cycle arrest at G1 for both breast cancer cell lines. The biosurfactant BioEG was shown to be more active than surfactin against the studied breast cancer cells. The results gathered in this work are very promising regarding the biosurfactants potential for breast cancer treatment and encourage further work with the BioEG glycoprotein.

## Introduction

Biosurfactants are molecules with surface activity produced by a number of microorganisms and that can be used in many biomedical applications (Gudiña et al. [2013]; Rodrigues et al. [2006a]; Rodrigues [2011]). Comprising a range of chemical structures, such as glycolipids, glycoproteins and lipopeptides, among others (Banat et al. [2010]), different biosurfactants are expected to exhibit diverse properties and physiological functions (Singh and Cameotra [2004]). Several researchers showed that biosurfactants partition at interfaces affecting the adhesion properties of microorganisms (Mireles et al. [2001]; Rivardo et al. [2009]; Rodrigues et al. [2004]; [2006b]; Velraeds et al. [1998]). Additionally, these molecules are able to increase membrane permeability by disrupting and lysing cell membranes (Heerklotz and Seelig [2006]; Lee et al. [2012]). This effect occurs due to changes in the physical membrane structure or by disrupting protein conformations, which changes important membrane functions (Ortiz et al. [2009]; Sánchez et al. [2010]; Van Hamme et al. [2006]; Zaragoza et al. [2009]).

Relevant properties of biosurfactants including their antimicrobial and antiviral activities, as well as their anti-adhesive activity against pathogens (Gudiña et al. [2010a]; [2010b]; Rodrigues [2011]) make them interesting alternatives for biomedical applications. Such molecules may be useful for gene transfection, as ligands for binding immunoglobulins, as adjuvants for antigens, as inhibitors of fibrin clot formation, as activators of fibrin clot lysis, but also as anti-adhesive biological coatings for prosthetic materials. The most well-known and studied biosurfactant is the lipopeptide surfactin, for which many efforts have been developed over the last years to prove its potential in many applications (Sen [2010]). Nevertheless, other less known and characterized biosurfactants could be more advantageous, especially if they have a broad spectrum of activity, and if their use with human cells is envisaged, since most of the surfactins that have been studied are hemolytic. Previously, we isolated and characterized a new biosurfactant produced by the probiotic bacterium *Lactobacillus paracasei* that could be potentially used with human cells (Gudiña et al. [2010a]; [2010b]). This biosurfactant is stable at 60°C and pH values ranging from 6.0 to 10.0; reduces the surface tension of water from 72.0 to 41.8 mN/m and has a critical micelle concentration of 2.5 mg/ml (Gudiña et al. [2010b]). Also, it presents antimicrobial activity against several microorganisms involved in diseases and infections in the urinary, vaginal and gastrointestinal tracts (Gudiña et al. [2010a]). The chemical composition of this biosurfactant (herein named BioEG) was studied and it was found to be a glycoprotein (Pinto et al. [2011]), which is in good agreement with the general composition reported for biosurfactants obtained from lactic acid bacteria (Brzozowski et al. [2011]; Golek et al. [2009]; Madhu and Prapulla [2013]; Moldes et al. [2013]; Tahmourespour et al. [2011a]; [2011b]).

One of the most thrilling results that have been recently reported for biosurfactants is their potential to act as anti-tumour agents interfering with some cancer progression processes (Fracchia et al. [2012]; Rodrigues [2011]). For example, glycolipids have been associated with growth arrest, apoptosis and differentiation of mouse malignant melanoma cells (Zhao et al. [1999]). Mannosylerythritol lipids showed pronounced growth inhibition and differentiation activities against human leukaemia cells (Isoda and Nakahara [1997]). Moreover, succinoyl trehalose lipids have been shown to inhibit growth and induce differentiation of HL60 human promyelocytic leukaemia cells (Sudo et al. [2000]) and human basophilic leukaemia cell line KU812 (Isoda et al. [1995]). Additionally, lipopeptides have also been widely studied for their potential anti-tumour activity. Several researchers reported the actions of surfactin and other lipopeptides against various cancer cell lines (Liu et al. [2010]; Seydlová and Svobodová [2008]; Sivapathasekaran et al. [2010]). Kim et al. ([2007]) evaluated the effect of surfactin on the human colon carcinoma cell line LoVo and showed that the lipopeptide presents a strong growth inhibitory activity by inducing apoptosis and cell cycle arrest. Lee et al. ([2012]) demonstrated that surfactin inhibited the growth of MCF7 human breast cancer cells in a dose-dependent manner. Moreover, Cao et al. ([2010]) showed that surfactin induced apoptosis of the same cells through a ROS/JNK-mediated mitochondrial/caspase pathway. The same authors also proven the cytotoxic effect of surfactin against the human chronic myelogenous leukaemia cells K562 and the hepatic carcinoma cells BEL7402 (2009a). Liu et al. ([2010]) evaluated the effect of lipopeptides by *Bacillus subtilis* HSO121 on Bcap-37 breast cancer cell lines and demonstrated that these compounds induced apoptosis in a dose-dependent manner. Furthermore, their results indicated that the disturbance of the cellular fatty acid composition of breast cancer cell lines, by lipopeptides, was related with apoptosis. In addition, several other lipopeptides (isoforms of surfactin and fengycin) were also found to have potent cytotoxic effects against the human colon cancer cell lines HCT15 and HT29 (Sivapathasekaran et al. [2010]). Since there is an enormous diversity of microbial surfactants, the attention of the scientific community in the search for new molecules with interesting anti-tumour activities is continuously increasing, as well as in looking deeply into their mechanisms of action.

In this work, the anti-tumour activity of a surfactin produced by *Bacillus subtilis* 573 and a glycoprotein (BioEG) produced by *Lactobacillus paracasei* subsp. *paracasei* A20 against two breast cancer cell lines, T47D and MDA-MB-231, was evaluated specifically regarding cell viability and proliferation. The biosurfactants’ effects on the viability of a non-tumour fibroblast cell line (MC-3 T3-E1) were also evaluated.

## Materials and methods

### Surfactin production and recovery

*Bacillus subtilis* strain 573 previously isolated and characterized in our lab (Gudiña et al. [2012]; Pereira et al. [2013]) was used to produce surfactin (strain deposited on Micoteca da Universidade do Minho (MUM) culture collection under the reference number MUM 14.01). The isolate was stored at -80°C in LB medium supplemented with 20% (v/v) glycerol solution. The composition of LB medium was (g l^-1^): NaCl 10.0; tryptone 10.0; yeast extract 5.0. The pH was adjusted to 7.0.

Surfactin production was conducted in 1 L flasks containing 400 ml of LB medium. The flasks were inoculated with 1% of a pre-culture grown in the same medium for 24 h. Cultures were incubated at 37°C for 72 h without agitation. At the end of the fermentation, cells were removed from the broth by centrifugation (10000 × g, 20 min, 20°C).

To recover the surfactin, cell-free supernatants were subjected to an acid precipitation as described elsewhere (Vaz et al. [2012]). Briefly, the supernatants were adjusted to pH 2.0 with HCl 6 M and left overnight at 4°C. Afterwards, the precipitate was collected by centrifugation (10000 × g, 20 min, 4°C) and washed twice with acidified water (pH 2.0). The precipitated biosurfactants were dissolved in a minimal amount of demineralized water and the pH was adjusted to 7.0 using NaOH 1 M. Finally, the biosurfactant solutions were freeze-dried and stored until further use.

Production and recovery of a glycoprotein biosurfactant from *Lactobacillus paracasei* subsp. *paracasei* A20.

*Lactobacillus paracasei* subsp. *paracasei* A20 isolated in a Portuguese dairy industry and previously reported as a biosurfactant-producing strain (Gudiña et al. [2010a]; [2010b]) was used to produce a glycoprotein herein named BioEG (Pinto et al. [2011]) (strain deposited on MUM culture collection under the reference number MUM 14.02). The strain was stored at −80°C in conventional synthetic MRSLac broth (standard MRS medium where glucose was replaced by lactose) containing 15% (v/v) glycerol solution.

For glycoprotein BioEG production in flasks, 600 ml MRSLac broth was inoculated with 1% of an overnight pre-culture grown in the same medium. The culture was incubated for 48 h at 37°C and 120 rpm. At the end of the fermentation, cells were harvested by centrifugation (10 000 × g, 5 min, 10°C), washed twice in demineralized water, and resuspended in phosphate-buffered saline (PBS: 10 mM KH_2_PO_4_/K_2_HPO_4_ and 150 mM NaCl with pH adjusted to 7.0) in a proportion 6:1. The bacteria were left at room temperature for 2 h with gentle stirring for biosurfactant release, as previously described (Gudiña et al. [2010b]). Subsequently, the bacteria were removed by centrifugation and the supernatant filtered through a 0.22 μm pore size filter. The supernatant was dialyzed against demineralized water at 4°C in a Cellu-Sep© membrane (cut-off 6000–8000 Da) and freeze-dried. The freeze-dried crude biosurfactant isolated from *L. paracasei* was then subjected to acidic precipitation as described elsewhere (Gudiña et al. [2010b]; Van Hoogmoed et al. [2000]). Briefly, the biosurfactant was resuspended in PBS (pH 7.0) to a concentration of 10 g l^-1^, and subsequently the pH was adjusted to 2.0 by adding HCl 1 M. The acidified solution was kept at 4°C for 2 h and the precipitate was collected by centrifugation (10000 × g, 15 min, 4°C) and washed twice with acidic water (pH 2.0). Afterwards the precipitate was dissolved in distilled water by adjusting the pH to 7.0 with NaOH 1 M, dialyzed against demineralized water at 4°C in a Cellu-Sep© membrane (cut-off 6000–8000 Da), freeze-dried and stored until further use.

### Cell lines

Breast cancer cell lines were kindly provided by the Institute of Molecular Pathology and Immunology of the University of Porto (IPATIMUP, Porto, Portugal). The MDA-MB-231 cell line was established from a pleural effusion obtained from a 51-yr-old female patient with breast cancer. Also, the T47D cell line was isolated from a pleural effusion obtained from a 54-yr-old female patient with an infiltrating ductal carcinoma of the breast. A non-tumour fibroblast cell line, mouse embryo fibroblast 3 T3 cells (MC-3 T3-E1), was used as a control. All cells were maintained in an incubator with a 5% CO_2_ atmosphere at 37°C. The culture medium used was the Dulbecco’s modified Eagle medium (DMEM; GIBCO, Invitrogen, Barcelona, Spain) supplemented with 10% fetal bovine serum (GIBCO, Invitrogen) and 1% penicillin/streptomycin (Invitrogen).

### Cell viability

Breast cancer cells were exposed to five concentrations of surfactin (0.05, 0.1, 0.2, 0.5 and 1.0 g l^-1^) and four of BioEG (0.05, 0.1, 0.15 and 0.2 g l^-1^). Biosurfactant stock solutions were prepared in PBS. Control experiments using the non-tumour MC-3 T3-E1 cell line were also conducted. The cell viability was determined using the MTS (3-(4,5-dimethylthiazol-2-yl)-5-(3-carboxymethoxyphenyl)-2-(4-sulfophenyl)-2H-tetrazolium) method. A commercial kit was used according to the manufacturer instructions (Promega, PROM G35800001, Lisbon, Portugal). In these experiments, 100 μl of cell suspension was added to each well of a 96-well plate. Additionally, control wells were included consisting of DMEM medium, PBS and the biosurfactants prepared in PBS at the concentrations under study. When a cell concentration of 1×10^5^ cells ml^-1^ was obtained, adequate volumes of the biosurfactant solutions were added to the wells and incubated for 24, 48 and 72 h. Afterwards, 20 μl of the Cell Titter 96 AQueous One Solution Cell Proliferation Assay reagent (MTS) was added to each well and left in the incubator (37°C, 5% CO_2_) for 2 h after which the cell viability was quantified by recording the absorbance at 490 nm.

For each biosurfactant and cancer cell line, a dose-response curve was generated and the growth inhibition of 50% (GI50), corresponding to the concentration of compound that inhibits 50% of the cell growth, was determined. The results are expressed as percentage of viable cells compared to the control and represent an average of 3 independent cultures with 3 wells per concentration in each experiment. Each exposure time was studied in triplicate with independent cultures.

### Cell cycle

Cells were grown in petri dishes (diameter 55 mm) until the total cell number reached 5×10^5^. Then, the medium was removed and replaced by fresh medium containing biosurfactants (0.05 g l^-1^ surfactin or 0.15 g l^-1^ BioEG). After 24 h (for surfactin) or 48 h (for BioEG), cells were trypsinized, washed with 5 ml PBS and finally centrifuged (1200 rpm, 5 min, 4°C). The cell pellet was resuspended in 500 μl PBS and kept in ice for 15 min. Cell suspension was readily mixed with 1.5 ml ethanol 96% at -20°C, and the final mixture was kept in ice during 15 min to allow cell fixation.

Afterwards, cell suspension was washed twice with PBS and resuspended in 500 μl of PBS. A volume of 50 μl of RNAse A (0.2 mg ml^-1^) was added to the mixture, vortexed and incubated at 37°C for 15 min. Subsequently, 60 μl of propidium iodide (PI) at 0.5 mg ml^-1^ was added to the previous mixture. The final mixture was again mixed in the vortex and kept in the dark for 30 min until analysis on a flow cytometer (Coulter Epics XL Flow Cytometer, Beckman Coulter Inc., Miami, FL, USA). The cell cycle analysis using the non-tumour cell line MC-3 T3-E1 was conducted only for the experiments with the BioEG biosurfactant. Three independent analyses were performed for each cell line and experimental condition. The flow cytometry results were analyzed with the software FlowJo version 7.6. (Tree Star, Inc., Ashland, OR, USA).

### Statistical analysis

A two-way ANOVA was used for the statistical evaluation of significant differences among the tested biosurfactant concentrations and exposure times as compared to the controls. Statistical analyses were performed in Microsoft Office Excel 2007 (Microsoft Corp., Redmond, WA).

## Results

In this work, the potential anti-tumour activity of two biosurfactants against two breast cancer cell lines was evaluated. Specifically, we used a surfactin produced by *B. subtilis* 573 isolated by our research group, and a glycoprotein (herein named BioEG) produced by *L. paracasei* subsp. *paracasei* A20 isolated in a Portuguese dairy industry.

### Effect of biosurfactants on cell viability

#### Surfactin

**Figure 1:**
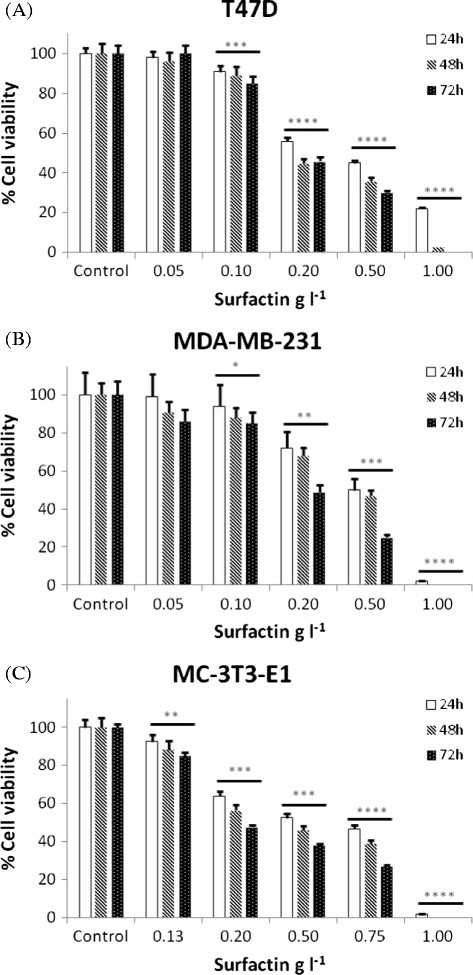
**Dose-response curve for T47D (A) and MDA-MB-231 (B) breast cancer and (C) MCT-3 T3-E1 non-tumour cell lines exposed to different concentrations of surfactin for 24, 48 and 72 h.** Values represent the average of 3 independent cultures with 3 replicates per concentration in each experiment. Each exposure time was studied in triplicate with independent cultures. All results were normalized and the results correspond to the mean ± standard deviation of three independent experiments. *P < 0.05; **P < 0.01; ***P < 0.005 and ****P < 0.001 when concentrations and exposure times were compared to the control.

Surfactin was tested in five concentrations for three exposure times against T47D and MDA-MB-231 breast cancer cell lines. The effect of the biosurfactant on a non-tumour cell line (MCT-3 T3-E1) was also evaluated. Figure 1 shows the dose-response curves obtained for all cell lines.

Positive controls (without surfactin) were included and correspond to 100% cell viability. Negative controls, containing only medium and surfactin at each studied concentration, were also included to eliminate any possible interference with the method.

The results obtained clearly show a decrease in the percentage of viable cells with increasing surfactin concentrations and exposure times, thus suggesting its cytostatic/cytotoxic effect against the studied breast cancer cell lines. However, for the highest surfactin concentration studied, a significant decrease of the total number of cells was observed, probably due to a prevalence of a detergent-like effect leading to cell membrane disruption (Heerklotz and Seelig [2006]; Kim et al. [2007]; Lee et al. [2012]). This effect was observed for the experiments in which cells were exposed to 1 g l^-1^ surfactin for 48 and 72 h.

Based on these findings, 0.5 g l^-1^ surfactin was found to be effective in decreasing T47D cell viability (Figure 1A) to values between 30 and 45% depending on the exposure time. The differences obtained for the range of surfactin concentrations evaluated as compared to the control were found to be statistically significant (P-value < 0.005), except for the lowest concentration (0.05 g l^-1^). Globally, the differences observed between exposure times (*F*-value = 5.2; *F crit* = 4.1) and concentrations (*F*-value = 180.0; *F crit* = 3.3) were found to be statistically significant.

For MDA-MB-231 cell line (Figure 1B), the conditions that promoted a more pronounced decrease of cell viability, without membrane disruption detectable by morphological visualization, were 24 h exposure to 0.5 g l^-1^ surfactin. Compared with the control, this concentration decreased MDA-MB-231 cell viability between 50 and 75% depending on the exposure time.

The differences obtained for all surfactin concentrations studied were statistically significant (P-value < 0.05), except for the lowest concentration. Moreover, the ANOVA results showed that the differences observed between exposure times (*F*-value = 6.1; *F crit* = 4.1) and concentrations (*F*-value = 118.6; *F crit* = 3.3) were significant. Furthermore, the concentration of surfactin that inhibits 50% of cell growth (GI50) for each exposure time and breast cancer cell line was determined. The GI50 values were found to be higher for MDA-MB-231 cells (24 h: GI = 0.50 ± 0.07; 48 h: GI = 0.45 ± 0.05; 72 h: GI = 0.20 ± 0.02) than for T47D (24 h: GI = 0.40 ± 0.03; 48 h: GI = 0.18 ± 0.02; 72 h: GI = 0.18 ± 0.01).

The same trends regarding surfactin concentrations and exposure times were observed in the experiments conducted with the non-tumor MCT-3 T3-E1 cell line (Figure 1C). Therefore, given the toxicity of surfactin against this cell line, no further cell cycle experiments were conducted.

#### BioEG

**Figure 2:**
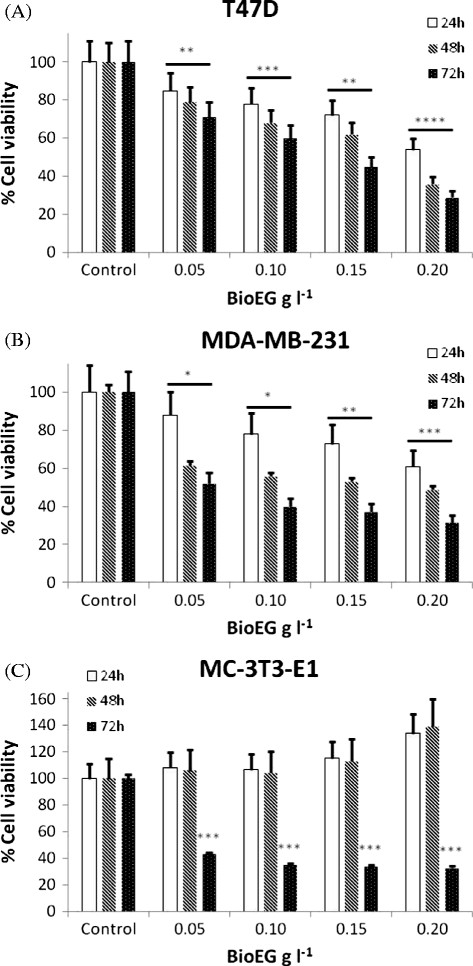
**Dose-response curve for T47D (A) and MDA-MB-231 (B) breast cancer and (C) MCT-3 T3-E1 non-tumour cell lines exposed to different concentrations of BioEG for 24, 48 and 72 h.** Values represent the average of 3 independent cultures with 3 replicates per concentration in each experiment. Each exposure time was studied in triplicate with independent cultures. All results were normalized and the results correspond to the mean ± standard deviation of three independent experiments. *P < 0.05; **P < 0.01; ***P < 0.005 and ****P < 0.001 when concentrations and exposure times were compared to the control.

Similarly, our biosurfactant BioEG was tested in four concentrations for three exposure times against the same breast cancer and control cell lines, as shown in Figure 2. Positive controls (without BioEG) and negative controls (medium and BioEG at each studied concentration) were also included.

The results gathered in Figure 2 clearly show that BioEG decreased cell viability of both breast cancer cell lines, an effect that is dependent on the biosurfactant concentration and exposure time. For the 24 h exposure time there is not a very pronounced effect on cell viability, especially at the lower biosurfactant concentrations. For the highest exposure times (48 and 72 h), the number of viable cells were found to decrease significantly. In addition, microscopic observation of the cell cultures showed that a 72 h exposure to the higher BioEG concentration (0.2 g l^-1^) led to a considerable amount of cell death, since only few viable cells were present in the sample after incubation under such conditions. These results suggest the prevalence of a detergent-like effect under these conditions.

A concentration of 0.15 g l^-1^ of BioEG was found to be effective in decreasing T47D cell viability (Figure 2A) to 72%, 62% and 45% for 24, 48 and 72 h exposure times, respectively. Comparing the different concentrations of BioEG with the control, statistical differences were found (*P*-value < 0.01). Moreover, different concentrations (*F*-value = 10.8; *F crit* = 4.5) and exposure times (*F*-value = 46.0; *F crit* = 3.8) were found to significantly influence the effect of BioEG.

Regarding the MDA-MB-231 cell line (Figure 2B), a decrease in the cell viability was also observed after incubating cells with 0.15 g l^-1^ of BioEG, leading to final values of 73%, 53% and 37% for an exposure time of 24, 48 and 72 h, respectively. Also in this case all the differences observed among different concentrations and exposure times were found to be statistically significant.

Comparing the effect of BioEG between the two cancer cell lines, it seems that MDA-MB-231 cells are more susceptible to the presence of the biosurfactant, since higher toxicities were found at lower concentrations. On the other hand, in the cytotoxicity studies conducted with surfactin, the MDA-MB-231 cells were found to be less susceptible than T47D. Interestingly, BioEG showed no effect on the viability of MCT-3 T3-E1 cells (Figure 2C); except for the longer exposure time (72 h) for which some cell lysis was observed. The differences observed among the several BioEG concentrations and exposure times (except for 72 h) were not statistically significant. Based on the cytotoxicity results this cell line was further used in the cell cycle experiments to evaluate the effect of BioEG on proliferation*.*

The GI50 for both cancer cell lines when exposed to BioEG was also determined and compared to the results obtained with surfactin. The GI50 values obtained for MDA-MB-231 cells were above 0.20 ± 0.03 (24 h); 0.20 ± 0.04 (48 h); and 0.06 ± 0.01 (72 h). For T47D, The GI values determined were above 0.20 ± 0.01 (24 h); 0.18 ± 0.02 (48 h); and 0.14 ± 0.01 (72 h). It is possible to observe that BioEG has a high anticancer potential, especially in MDA-MB-231 cells.

### Cell cycle

#### Surfactin

The evaluation of cell proliferation through analysis of cell cycle was performed by flow cytometry for both cell lines subjected to a 24 h exposure to 0.05 g l^-1^ surfactin. As shown in Table 1, surfactin induced a G1 arrest in both cell lines with the concomitant decrease of cells that were synthesizing DNA (cells at S phase). Contrary to the MTS results (Figure 1), the effect was stronger in MDA-MB-231 cells. These data show that surfactin inhibits the synthesis of DNA, therefore negatively influencing the cell proliferation. Although further research is required to elucidate the surfactin targets within the cell, these results clearly demonstrate that the lipopetide can trigger cell cycle arrest at the G1 phase; contrarily to the results previously reported by Cao et al. ([2009a]) that observed a G2/M arrest.

**Table 1:** **Effect of a 24 h exposure to 0.05 g l**^**-1**^**surfactin on the cell cycle of T47D and MDA-MB-231 breast cancer cell lines**

**Sample**	**% Cells in different phases of the cell cycle**		
	**G0-G1**	**S**	**G2-M**
T47D – control	49.5 ± 6.3	32.9 ± 3.6	17.6 ± 0.75
T47D – surfactin	51.4 ± 0.2	30.8 ± 1.1	17.6 ± 2.3
MDA-MB-231 – control	44.3 ± 0.6	28.3 ± 16.0	27.4 ± 1.8
MDA-MB-231 – surfactin	55.2 ± 20.4	19.1 ± 5.0	25.8 ± 15.8

Results correspond to the average percentage of cells in the difference cell cycle phases from three independent assays ± stdev.

#### BioEG

Similarly, Table 2 shows the results of the effect of 0.15 g l^-1^ BioEG in cell cycle of T47D and MDA-MB-231 cells after 48 h exposure. Like surfactin, BioEG also induced a G1 arrest in both cell lines associated with a decrease of cells at S and G2 phases. However, the effect was stronger in the T47D cell line, contrary to what occurred in the MTS results. The discrepancy between BioEG and surfactin effectiveness in cell cycle arrest may be the result of different mechanisms of action being in place in different cell lines. Additionally, BioEG did not affect significantly the cell cycle of MC-3 T3-E1 cells (Table 3).

**Table 2:** **Effect of a 48 h exposure to 0.15 g l**^**-1**^**BioEG on the cell cycle of T47D and MDA-MB-231 breast cancer cell lines**

**Sample**	**% Cells in different phases of the cell cycle**		
	**G0-G1**	**S**	**G2-M**
T47D – control	41.1 ± 7.0	36.4 ± 4.0	22.3 ± 3.5
T47D – BioEG	48.6 ± 0.8	29.6 ± 1.39	21.5 ± 0.21
MDA-MB-231 – control	58.1 ± 11.0	20.0 ± 3.2	22.0 ± 4.3
MDA-MB-231 – BioEG	61.3 ± 7.1	16.4 ± 6.3	20.9 ± 5.7

Results correspond to the average percentage of cells in the difference cell cycle phases from three independent assays ± stdev.

**Table 3:** Effect of a 48 h exposure to 0.15 g l^-1^ BioEG on the cell cycle of MC-3 T3-E1 cell line

**Sample**	**% Cells in different phases of the cell cycle**		
	**G0-G1**	**S**	**G2-M**
MC-3 T3-E1 – control	51.4 ± 4.0	23.7 ± 1.3	24.8 ± 1.0
MC-3 T3-E1 – BioEG	46.9 ± 4.1	26.3 ± 8.0	27.4 ± 3.0

Results correspond to the average percentage of cells in the difference cell cycle phases from three independent assays ± stdev.

## Discussion

Biosurfactants are versatile molecules that have been raising the interest of many industrial fields, including for biomedical applications. The use of these molecules as anti-tumour agents is still unexploited although there is now some evidence of their potential. In this work, a surfactin produced by *Bacillus subtilis* 573 and a glycoprotein (BioEG) produced by *Lactobacillus paracasei* subsp. *paracasei* A20 were evaluated for their effect on breast cancer cells viability and proliferation. Two breast cancer cell lines, T47D and MDA-MB-231, and a non-tumour fibroblast cell line (MC-3 T3-E1) were used. Surfactin was shown to have a cytotoxic effect against T47D and MDA-MB-231 breast cancer cells, which is in accordance with other studies reported in the literature using other types of cancer cells (Cao et al. [2009a]; [2010]; Kim et al. [2007]; Lee et al. [2012]; Sivapathasekaran et al. [2010]). It is well-known that cells are very sensitive to this lipopeptide mainly due to its effect on the cell membrane, thus low concentrations are required to observe an effect on cell viability. As seen in the current work, higher surfactin concentrations lead to cell death due to membrane disruption, which is not desirable. Therefore, the amounts of surfactin to be used need to be carefully evaluated.

Hwang et al. ([2007]) showed that the LD50 (dose of surfactin necessary to cause death of 50% of the test population) for surfactin administration in mice is above 100 mg Kg^-1^. Also, the authors reported that the administration of 10 mg of surfactin for a long period of time did not reveal signs of apparent toxicity.

A drawback on the use of surfactin as a chemotherapeutic agent is its hemolytic activity (Dehghan-Noudeh et al. [2005]) that has been reported for concentrations above 0.05 g l^-1^. From our results (Figure 1), this surfactin concentration exerts a low cytotoxic effect in the cancer cells under study. Nevertheless, to guarantee the non-occurrence of hemolysis, a 24 h exposure to this surfactin concentration was chosen as the most adequate binomial concentration/exposure time for the cell cycle experiments.

Since surfactin has never been tested in humans, to prevent future complications several strategies have been explored envisaging its use as a safe therapeutic agent. Symmank et al. ([2002]) reported several minor modifications of the surfactin molecule by altering surfactin synthetase. These modifications changed the molecule toxicity profile, resulting in a “new” lipopeptide with improved activity and not revealing any signs of toxicity or hemolytic activity. Another interesting approach consists in the incorporation of surfactin in nanoparticles in order to provide a directed administration and *in situ* release of the cyclic peptide.

Surfactin is an amphipathic molecule that has been reported as a plasma membrane destabilization agent, thus disturbing its integrity (Sánchez et al. [2010]). The key step of such process is the integration of the surfactin molecule in the lipid bilayer, inducing modifications through the formation of pores or channels, or through its detergent activity. The lipopeptide penetrates the membrane by hydrophobic interactions influencing the order of the hydrocarbon chains and consequently the membrane thickness. After this first interaction, the cyclic peptide suffers conformational modifications that cause the dehydration of the phospholipid polar group (Carrillo et al. [2003]). These structural perturbations are directly linked to the lipopeptide concentration. According to Fracchia et al. ([2010]), at low concentrations, surfactin penetrates rapidly on the cellular membrane, forming micelles together with phospholipids. At moderate concentrations surfactin can induce pore formation on the lipid bilayers, and at high concentrations the detergent-like effect prevails resulting in total membrane loss. The detergent-like effect of surfactin (Heerklotz and Seelig [2006]; Lee et al. [2012]; Kim et al. [2007]) and other biosurfactants (Burgos-Díaz et al. [2013]; Janek et al. [2013]) has been reported by several authors.

Cao et al. ([2010,2011]) demonstrated that surfactin induces apoptosis in human breast cancer MCF7 cells through a ROS/JNK-mediated mitochondrial/caspase pathway. Also, the same research group demonstrated the cytotoxic effect of surfactin, in a dose-dependent manner, against the human chronic myelogenous leukaemia cells K562 and the hepatic carcinoma cells BEL7402 (Cao et al. [2009a]). As far as we know, this is the first study on the effect of surfactin against T47D and MDA-MB-231 breast cancer cell lines.

Regarding the BioEG biosurfactant, the results showed that it presents a high anticancer potential, especially in MDA-MB-231 cells. Besides being more susceptible to BioEG, the MDA-MB-231 cell line was also found to be less prone to cell lysis comparing to T47D, since under the microscope the number of MDA-MB-231 intact cells at 72 h exposure to 0.2 g l^-1^ BioEG was higher. The breast cancer cell lines used in this work have different features that may be responsible for the different behaviors observed when exposed to BioEG. The most important difference between these two cell lines is the absence of estrogen receptor (ER) in MDA-MB-231 cells. Additionally, the ER negative cell line also lacks E-cadherin and desmoplaquin I/II contrarily to the T47D cell line (Sommers et al. [1994]). On the other hand, vimentin is present in MBA-MB-231 cells contrary to T47D, thus giving the ER negative cell line a much more invasive profile (Sommers et al. [1994]). Data collected in the current work, both with surfactin or BioEG, are very promising since ER negative cell lines (e.g. MDA-MB-231) are not susceptible to regular chemotherapeutic agents such as Tamoxifen (Burdall et al. [2003]; O’Lone et al. [2004]; Sommers et al. [1994]), and therefore any new alternative agents that can have an effect on such cells are extremely useful. ER is associated to cell growth regulation, differentiation and maintenance of cell balance (O’Lone et al. [2004]) and its function is dependent on estrogen binding. Desmoplaquins type I and II are molecules associated to E-cadherin, whose expression is altered in some tumours, which influences cell adhesion and migration (O’Lone et al. [2004]). The absence of these molecules confers the cancer cells the ability to migrate and consequently to form metastasis (Von Schlippe et al. [2000]). Vimentin is another key molecule that is frequently altered in cancer cells, and is responsible for cell anchorage and regulation of organelle position within the cytosol. The presence of such molecule is an indicator of a high invasive ability and also a high resistance to chemotherapeutics. As mentioned above, both biosurfactants were found to be active against MDA-MB-231 cells, which suggest that these compounds can be an effective alternative to the chemotherapeutics currently used.

To our knowledge, this is the first report on the use of BioEG against cancer cell lines and there are also no reports on the toxicity of this biosurfactant against non-tumour cells. Interestingly, we found that the exposure of the non-tumour MC-3 T3-E1 fibroblast cell line up to 48 h to several concentrations of BioEG (up to 0.2 g l^-1^) did not affect cell viability or even promoted it slightly, which shows some specificity of this glycoprotein to cancer cells. Although very promising, this result is still preliminary and further experiments using non-tumour breast epithelial cells should be conducted.

In order to get more insights on the effect of the biosurfactants against breast cancer cell lines, cell cycle analysis was performed at conditions that cell lysis was found to be residual. Both surfactants induced a G1 arrest and decreased DNA synthesis, which demonstrate their ability to affect cell cycle progression and thus to inhibit cell proliferation. With these results it is possible to speculate about some mechanisms of action. At the G1 phase of the cell cycle, a set of molecular events occur that can lead cells to cease their progression in the cell cycle, and therefore stop dividing themselves. The G1 checkpoint is responsible for the G1 arrest, for example in response to DNA damage, and it is intimately related with the activity of the p53 gene (Xiao et al. [2004]). However, both breast cancer cell lines used in this work possess a mutation on the p53 gene, a transcriptional regulation factor that can also work as a transcriptional repressor. Another important molecule present at G1 checkpoint is the protein p16, an inhibitor of the retinoblastoma protein (Rb) phosphorylation (Xiao et al. [2004]). For the cell to progress to the S phase, Rb needs to be phosphorylated by the cyclin-dependent kinases (CDK). In mammalian cells, cyclin D and E form active complexes with CDKs in order to promote cell proliferation. Whenever an anti-proliferative stimulus is present these cyclins are downregulated. All these key molecules can be targets of the biosurfactants used, but it is important to notice that at this checkpoint the cell can also stop proliferating due to entrance in a latency phase, the so-called G0 phase. At the G0 phase of the cell cycle, a transcriptional factor (E2F) is connected to the hypo-phosphorylated form of Rb (active form), thus preventing the cell to undergo the cell cycle (Xiao et al. [2004]).

Cao et al. ([2009b,^2011^]) reported that surfactin produced by *Bacillus subtilis natto* TK-1 negatively influences the cell cycle progression of MCF7 breast cancer cells. In that study, surfactin was found to decrease about 22.2% the G1 phase, the S phase remained constant and an increase of 16.6% of the G2 phase was observed (Cao et al. [2009b]). Cell proliferation was found to be inhibited through cell arrest at G2/M phase. Western blot revealed that surfactin induced accumulation of the tumour suppressor p53 and cyclin kinase inhibitor p21^waf1/cip1^, and inhibited the activity of the G2-specific kinase, cyclin B1/p34^cdc2^. Based on their results the authors suggest that the mechanism by which surfactin caused G2/M arrest is through cell cycle factor regulation. Since the cell line used by these authors was different from the ones evaluated in the current work, particularly in what regards the p53 gene status, it is not possible to perform a straightforward comparison of the results. Nevertheless, the same trend regarding a decrease in cell proliferation could be observed. As previously mentioned, the same authors (Cao et al. [2010]) demonstrated that surfactin induces apoptosis of MCF7 cells through a ROS/JNK-mediated mitochondrial/caspase pathway. Also, surfactin was found to induce ROS formation leading to the mitochondria permeability transition pore (MPTP) opening together with the collapse of mitochondrial membrane potential which in turn promoted the increase of cytoplasmic Ca^2+^ concentration (Cao et al. [2011]). Further, cytochrome c was released from mitochondria to cytoplasm through the MPTP which activated caspase-9, eventually inducing apoptosis. Our results suggest that, in the conditions used, surfactin does not affect apoptosis of the studied breast cancer cell lines, since no increase in the fraction of cells at sub-G1 phase could be observed (*data not shown)*, which is typical of apoptosis induction. This may be attributable to the mutation of the p53 gene present in both studied breast cancer cell lines, which is known to confer resistance to death by apoptosis (Xavier et al. [2012]). In summary, in this work we found that surfactin, when used in adequate concentrations and exposure times, does not cause membrane disruption but decreases cellular proliferation. It is important to notice that the biosurfactant BioEG, a glycoprotein produced by *L. paracasei subsp. paracasei* A20, has been isolated and characterized by our group (Gudiña et al. [2010b]; Pinto et al. [2011]), so there are no previous reports on its potential anti-tumour activity. Therefore, it will be interesting in future studies to explore more in detail the mechanisms of action of BioEG since besides being non-toxic to non-tumour cells, in some circumstances, it presented higher anti-tumour activity as compared to surfactin.

Interestingly, the biosurfactant BioEG seems to have a more secure profile when exposed to non-tumour cells. Besides not affecting cell viability up to 48 h of exposure, BioEG did not affect significantly the cell cycle of fibroblasts (MC-3 T3-E1 cells). Contrary to breast cancer cells, there was a small increase of cells at S phase, which may indicate a promotion of cell proliferation in view of the higher number of cells synthesizing DNA. These results represent an important finding as it seems that BioEG not only can be used as a chemotherapeutic agent, but also in many other biomedical applications, such as invasive surgery recovery. BioEG proved to selectively affect the two breast cancer cell lines under study, and although the non-tumour cells (control cells) are not from human breast, the results are promising and open the possibility of using the biosurfactant on tissue regeneration.

Currently, the studies explaining the mechanisms by which biosurfactants exert their anti-tumour activity are scarce. Nevertheless, from this work it was found that these molecules action is not limited to their effect on cell membranes (membrane disruption), although it is still unclear if biosurfactants can be internalized by cells or if they act via modulating signalling transduction by binding to membrane receptors or by disrupting anchorage of signalling proteins on membrane lipid domains. Moreover, it is reasonable to expect that breast cancer cells present different response profiles when exposed to biosurfactants due to their different molecular signatures. However, no significant differences could be found between cancer cells with or without estrogen receptor, so we can suggest that the biosurfactants activity is not related with the presence of such receptor.

In summary, the results gathered in this work are very encouraging regarding the biosurfactants potential for breast cancer treatment and even for other biomedical applications; nevertheless further research on their mechanisms of action is required.

## Competing interests

The authors declare that they have no competing interests.

## Authors’ contributions

CD carried out the cytotoxicity and cell proliferation studies, and participated in the interpretation and discussion of the results. EJG carried out the production of the biosurfactants and helped to draft the manuscript. CFL participated in the design of the study, in the analysis and discussion of the cell cycle experiments, and helped to draft the manuscript. LRR conceived of the study, participated in its design and coordination, performed the statistical analysis and drafted the manuscript. All authors read and approved the final manuscript.
